# 2026 Pregnancy Meeting™ Oral Abstracts

**DOI:** 10.1002/pmf2.70189

**Published:** 2026-02-09

**Table:** 

Wednesday, February 11, 2026 8:00 AM – 10:00 AM		
Time	Abstract	Oral Plenary Session 1
8:00 AM	1	**A Randomized Controlled Trial of Baby2Home: A Postpartum Digital Mental Health Intervention**
		**Emily S. Miller^1^**; Jacqueline Gollan^2^; Lutfiyya N. Muhammad^3^; Kathleen O'Sullivan^2^; Dinah Williams^4^; Malika D. Shah^3^; Lynn M. Yee^3^; Siyuan Dong^3^; Young S. Lee^3^; Craig F. Garfield^2^ ^1^Women & Infants Hospital of Rhode Island/Warren Alpert Medical School of Brown University, Providence, RI; ^2^Northwestern University Feinberg School of Medicine, Evanston, IL; ^3^Northwestern University Feinberg School of Medicine, Chicago, IL; ^4^Women & Infants Hospital of Rhode Island, Providence, RI
8:15 AM	2	**Glycemic Measures in Early Pregnancy and Neonatal Outcomes**
		**Noelia Zork^1^**; Alan Kuang^2^; Patrick M. Catalano^3^; Francesca Facco^4^; Maisa Feghali^5^; William A. Grobman^6^; Erin S. LeBlanc^7^; William L. Lowe^8^; Audrey Merriam^9^; Mirella Mourad^10^; Caryn E.S. Oshiro^11^; Camille E. Powe^12^; Uma M. Reddy^1^; Dwight J. Rouse^13^; Jennifer Sherr^14^; Alexandra C. Spadola^15^; Kimberly K. Vesco^7^; Lynn M. Yee^16^; Denise Scholtens^16^; Erika F. Werner^15^ ^1^Columbia University Irving Medical Center, New York, NY; ^2^Northwestern University, Chicago, IL; ^3^MGH REU, Boston, MA; ^4^UPMC, UPMC, PA; ^5^Magee Women's Research Institute, University of Pittsburgh, Pittsburgh, PA; ^6^Warren Alpert Medical School of Brown University, Providence, RI; ^7^Kaiser Permanente Center for Health Research, Portland, OR; ^8^Northwestern University, Northwestern University, IL; ^9^Yale School of Medicine, Yale School of Medicine, CT; ^10^Division of Maternal‐Fetal Medicine, Department of Obstetrics and Gynecology, Columbia University Irving Medical Center, New York, NY; ^11^Kaiser Permanente Hawaii, Center for Integrated Health Care Research, Honolulu, HI; ^12^Massachusetts General Hospital, Boston, MA; ^13^Women & Infants Hospital of Rhode Island/Warren Alpert Medical School of Brown University, Providence, RI; ^14^Yale School of Medicine, New Haven, CT; ^15^Tufts University School of Medicine, Boston, MA; ^16^Northwestern University Feinberg School of Medicine, Chicago, IL
8:30 AM	3	**Randomized Trial of Bovine Lactoferrin for Treatment of Iron Deficiency in Pregnancy**
		**Joanne M. Said^1^**; Rachel L. O'Connell^2^; Emily Callander^3^; Reynolds M. Rebecca^4^; Michael Dibley^3^; Sajid Soofi^5^; Sidrah Nausheen^5^; Karen M. Baker^6^; Amanda Henry^7^; Raiyomand Dalal^8^; Josephine Laurie^9^; Stephen Cole^10^; Lourdes St George^11^; Amanda Dennis^12^; Christos Georgiou^13^; Irena Idel^14^; Jonathan M. Morris^15^; Valeria Lanzarone^16^; Ben W. Mol^17^; William Tarnow‐Mordi^2^ ^1^Western Health/University of Melbourne, University of Melbourne, Victoria; ^2^NHMRC Clinical Trials Centre, University of Sydney, Camperdown, New South Wales; ^3^University of Technology Sydney, Sydney, New South Wales; ^4^University of Edinburgh, Edinburgh, Scotland; ^5^Aga Khan University Hospital, Aga Khan University Hospital, Sindh; ^6^Royal Brisbane and Women's Hospital, Royal Brisbane and Women's Hospital, Queensland; ^7^The George Institute for Global Health, UNSW, Sydney, New South Wales; ^8^Campbelltown Hospital, Campbelltown, New South Wales; ^9^Mater Misericordiae, Brisbane, Queensland; ^10^Epworth Healthcare, East Melbourne, Victoria; ^11^Auburn Hospital, Auburn, New South Wales; ^12^Launceston General Hospital, Launceston General Hospital, Tasmania; ^13^Angliss Hospital, Upper Ferntree Gully, Victoria; ^14^Box Hill Hospital, Box Hill, Victoria; ^15^Royal North Shore, Sydney, New South Wales; ^16^Nepean Hospital, Penrith, New South Wales; ^17^Monash Health, Monash University, Victoria

**Table:** 

Wednesday, February 11, 2026 8:00 AM – 10:00 AM		
Time	Abstract	Oral Plenary Session 1, continued
8:45 AM	4	**Performance of a Single Gene Non‐Invasive Prenatal Screening Test for Recessive Conditions**
		**Rajeevi Madankumar^1^**; Thomas Westover^2^; Luis D. Pacheco^3^; Mollie McDonnold^4^; Jessica Scholl^5^; Lawrence D. Platt^6^; Ravindu P. Gunatilake^7^; Olaide Ashimi Balogun^8^; Angela T. Bianco^9^; Laura G. Hendon^10^; Todd Rosen^11^; Ashley S. Roman^12^; Karen Fitch^13^; Karin Niemeyer^13^; Ravi Vijaya Satya^13^; Ebad Ahmed^13^; Xingwu Lu^13^; Wenbo Xu^13^; Hyunseok P. Kang^13^; Fergal Casey^13^; Matthew Rabinowitz^13^; Emily Morton^13^; Vivienne Souter^13^; Jeffrey T. Meltzer^13^; John Williams, III^14^ ^1^Northwell Health, New Hyde Park, NY; ^2^Capital Health, Pennington, NJ; ^3^University of Texas Medical Branch, Galveston, TX; ^4^St. David's Women's Center of Texas, Austin, TX; ^5^Weill Cornell Medicine, New York, NY; ^6^UCLA David Geffen School of Medicine and Cedars‐Sinai Medical Center, Los Angeles, CA; ^7^Valley Perinatal Services, Chandler, AZ; ^8^Obstetrix Maternal‐Fetal Medicine Specialist of Houston, Houston, TX; ^9^Icahn School of Medicine at Mount Sinai, New York, NY; ^10^University of Mississippi Medical Center, Jackson, MS; ^11^Rutgers Robert Wood Johnson Medical School, New Brunswick, NJ; ^12^Division of Maternal‐Fetal Medicine, Department of Obstetrics and Gynecology, NYU Grossman School of Medicine, New York, NY; ^13^Natera, Inc., Austin, TX; ^14^Cedars‐Sinai Medical Center, Los Angeles, CA
9:00 AM	5	**A Maneuver to Decrease Perineal Lacerations and OASIS: Patient Positioning for Perineal Protection (4P) Study**
		**Marti D. Soffer^1^**; Kaitlyn E. James^2^; Pichliya Liang^3^; Arlin Delgado^2^; Matthew H. Mossayebi^2^; Margaret Hamp^3^; Molly R. Siegel^4^; Mark A. Clapp^5^; Anjali J. Kaimal^6^; William H. Barth, Jr.^1^ ^1^Department of Obstetrics and Gynecology, MassGeneral Brigham; Division of Maternal‐Fetal Medicine; Harvard Medical School, Massachusetts General Hospital, Boston, MA; ^2^Department of Obstetrics and Gynecology, MassGeneral Brigham; Division of Maternal‐Fetal Medicine; Harvard Medical School, Boston, MA; ^3^Department of Obstetrics and Gynecology, MassGeneral Brigham, Massachusetts General Hospital, Boston, MA; ^4^Massachusetts General Hospital, Division of Maternal Fetal Medicine, Boston, MA; ^5^Department of Obstetrics and Gynecology, Mass General Brigham, Massachusetts General Hospital, MA; ^6^Department of Obstetrics and Gynecology, University of South Florida, Tampa, FL
9:15 AM	6	**Fetal Growth Restriction: Comparison of Two National Surveillance Guidelines (FAME)**
		**Aaron W. Roberts^1^**; Mar Romero‐Lopez^2^; John W. Hotra^2^; Eleazar E. Soto^3^; Angela Glaser^4^; Robyn Garcia^2^; Jennie Coselli^2^; Suneet P. Chauhan^5^; Hector Mendez‐Figueroa^6^ ^1^Department of Obstetrics, Gynecology and Reproductive Sciences, McGovern Medical School at UTHealth Houston, Houston, TX; ^2^McGovern Medical School at UTHealth, Houston, TX; ^3^University of Texas Health Science Center in Houston, McGovern Medical School, Houston, TX; ^4^UT Houston, Houston, TX; ^5^Delaware Center for Maternal‐Fetal Medicine of Christiana Care, Newark, DE; ^6^Division of Maternal‐Fetal Medicine, Department of Obstetrics, Gynecology and Reproductive Sciences, McGovern Medical School at UTHealth Houston, Houston, TX
9:30 AM	7	**Randomized Trial of Cervical Pessary or Progesterone vs Placebo for Short Cervix in Twin Gestations**
		**Joseph R. Biggio Jr**. On behalf of the Eunice Kennedy Shriver National Institute of Child Health and Human Development (NICHD) Maternal‐Fetal Medicine Units (MFMU) Network, Bethesda, MD Ochsner Health, New Orleans, LA
9:45 AM	8	**Association Between SARS‐CoV‐2 Vaccine in Pregnancy and Child Neurodevelopment at 18‐30 Months**
		**George R. Saade^1^**; Brenna L. Hughes^2^ On behalf of the Eunice Kennedy Shriver National Institute of Child Health and Human Development Maternal‐Fetal Medicine Units Network, Bethesda, MD ^1^Eastern Virginia Medical School at Old Dominion University, Norfolk, VA; ^2^Department of Obstetrics and Gynecology, Duke University School of Medicine, Duke, NC

**Table:** 

Wednesday, February 11, 2026 10:00 AM – 10:30 AM		
Time	Abstract	Late‐Breaking Abstract Presentations Session 1
**10:00 AM**	**LB01**	**Single‐Dose Intravenous Iron vs Oral Iron for Postpartum Anemia: A Multi‐Center Randomized Controlled Trial (PRIORITY)**
		Richard Derman^1^; Shiva Goudar^2^; Mrityunjay C. Metgud^2^; Janet Moore^3^; Denise Babineau^3^; Elizabeth M. McClure^3^; **Rupsa C. Boelig** ^4^ NICHD Global Network for Women's and Children's Health Research, on behalf of the PRIORITY Trial Group ^1^Thomas Jefferson University, Philadelphia, PA; ^2^Jawaharlal Nehru Medical College, KLE Academy Higher Education and Research, Belagavi, Karnataka; ^3^RTI, Research Triangle Park, NC; ^4^Sidney Kimmel Medical College, Thomas Jefferson University, Philadelphia, PA
**10:15 AM**	**LB02**	**Universal Aspirin Administration for Prevention of Preeclampsia**
		**Elaine L. Duryea** ^1^; Anne L. Ambia^1^; Jessica E. Pruszynski^1^; Joyce Miller^1^; Sharon L. Hubbard^2^; Carrie Berge^2^; David B. Nelson^1^; Catherine Y. Spong^1^ ^1^University of Texas Southwestern Medical Center, Dallas, TX; ^2^Parkland Health, Dallas, TX

**Table:** 

Wednesday, February 11, 2026 1:00 PM – 3:30 PM		
Time	Abstract	Oral Concurrent Session 1 – Medical and Surgical Complications of Pregnancy
1:00 PM	9	**Comparative Effectiveness of Two Enhanced Prenatal Care Models on Mental Health: Randomized Trial**
		**Miriam Kuppermann^1^**; Jennifer N. Felder^2^; Daisy León‐Martínez^3^; Deborah Karasek^4^; Venise Curry^5^; Kristin Carraway^5^; Bethany Simard^6^; Kimberly Coleman‐Phox^7^; Brittany D. Chambers Butcher^8^; Patience A. Afulani^9^; Bridgette Blebu^10^; Cinthia Blat^7^; Mary A. Garza^5^; Charles E. McCulloch^11^ ^1^Department of Obstetrics, Gynecology and Reproductive Sciences, University of California, San Francisco, University of California San Francisco, CA; ^2^Department of Psychiatry and Behavioral Sciences, University of California, San Francisco, San Francisco, CA; ^3^Division of Maternal‐Fetal Medicine at the University of California San Francisco, University of California San Francisco, CA; ^4^Oregon Health & Science University‐Portland State University School of Public Health, Portland, OR; ^5^Central Valley Health Policy Institute at California State University, Fresno, Fresno, CA; ^6^Department of Obstetrics, Gynecology and Reproductive Sciences, University of California, San Francisco, UCSF, CA; ^7^Department of Obstetrics, Gynecology and Reproductive Sciences, University of California, San Francisco, San Francisco, CA; ^8^Department of Human Ecology, College of Agricultural and Environmental Sciences, University of California, Davis, Davis, CA; ^9^Department of Obstetrics, Gynecology and Reproductive Sciences, University of California, San Francisco, Universiy of California San Francisco, CA; ^10^Lundquist Institute for Biomedical Innovation ‐ Harbor‐UCLA, Los Angeles, CA; ^11^University of California, San Francisco, University of California San Francisco, CA
1:15 PM	10	**Hemoglobin Equilibration After Transfusion in Postpartum Patients**
		**Ellen H. Crowe^1^**; Ahmed Zaki Moustafa^2^; Megan C. Shepherd^3^; Baha M. Sibai^3^; Sean C. Blackwell^3^; Aaron W. Roberts^1^ ^1^Department of Obstetrics, Gynecology and Reproductive Sciences, McGovern Medical School at UTHealth Houston, Houston, TX; ^2^Division of Maternal‐Fetal Medicine, Department of Obstetrics, Gynecology and Reproductive Sciences, McGovern Medical School at UTHealth Houston, University of Texas ‐ Houston, TX; ^3^Division of Maternal‐Fetal Medicine, Department of Obstetrics, Gynecology and Reproductive Sciences, McGovern Medical School at UTHealth Houston, Houston, TX
1:30 PM	11	**Optimizing Iron Deficiency Management to Reduce Maternal Anemia and Peri‐Delivery Transfusion: A QI Initiative**
		**Carolyn M. Webster^1^**; Charlotte B. McCarley^2^; Gabriella D. Cozzi‐Glaser^3^; Jori May^4^; Amy Boone^5^; Ashley N. Battarbee^6^ ^1^University of Alabama at Birmingham, Birmingham, AL; ^2^University of Tennessee Medical Center, Knoxville, TN; ^3^Feinberg School of Medicine, Chicago, IL; ^4^University of Alabama at Birmingham, University of Alabama at Birmingham, AL; ^5^Indiana University, Indianapolis, IN; ^6^Center for Research in Women's Health, University of Alabama at Birmingham, Birmingham, AL

**Table:** 

Wednesday, February 11, 2026 1:00 PM – 3:30 PM		
Time	Abstract	Oral Concurrent Session 1 – Medical and Surgical Complications of Pregnancy, continued
1:45 PM	12	**Association Between Uterine Artery Doppler Velocimetry, Placenta Accreta Spectrum, and Degree of Placental Invasion**
		**Elias Kassir^1^**; Edgar A. Hernandez‐Andrade^2^; Donatella Gerulewicz^2^; Sarah T. Mehl^2^; Eleazar E. Soto^1^; Ramesha Papanna^1^; Sean C. Blackwell^2^; Baha M. Sibai^2^; Farah H. Amro^2^ ^1^University of Texas Health Science Center in Houston, McGovern Medical School, Houston, TX; ^2^Division of Maternal‐Fetal Medicine, Department of Obstetrics, Gynecology and Reproductive Sciences, McGovern Medical School at UTHealth Houston, Houston, TX
2:00 PM	13	**Investigating the Relationship Between Prenatal Methadone Exposure and Neonatal Withdrawal Using Cord Tissue Analysis**
		**Courtney Townsel^1^**; Makeda Turner^2^; Fatemeh Abdollah Biroon^2^; Vedavalli Govindan^3^; Eric K. Broni^4^; Jeannie C. Kelly^5^ ^1^University of Maryland School of Medicine, University of Maryland School of Medicine, MD; ^2^University of Maryland School of Medicine, Baltimore, MD; ^3^Campbell University School of Osteopathic Medicine, Lillington, NC; ^4^Yale University, New Haven, CT; ^5^Washington University School of Medicine, Washington University School of Medicine, MO
2:15 PM	14	**Role of Iron Supplementation in Pregnant Women With Non‐Anemic Iron Deficiency: A Randomized Controlled Trial**
		**Bijal Parikh^1^**; Mayi Gnofam^2^; Rakasa Pattanaik^3^; Brynn S. Franz^4^; Tiffany Yang^5^; Emily Stetler^2^; Chaitali Korgaonkar‐cherala^6^; Heidi Preis^5^; David J. Garry^7^; Cassandra Heiselman^8^; Kimberly Herrera^8^ ^1^St. Peters University Hospital, Warren, NJ; ^2^Stony Brook University, New York, NY; ^3^Beth Israel Deaconess Medical Center, Boston, MA; ^4^University of South Carolina School of Medicine ‐ ‐ Columbia, SC, East Northport, NY; ^5^Stony Brook University, Stony Brook, NY; ^6^Stony Brook Medicine, Stony Brook, NY; ^7^Stony Brook Medicine, Stony Brook Medicine, NY; ^8^Stony Brook University Hospital, Stony Brook, NY
2:30 PM	15	**Goal Maternal Hemoglobin in 2nd Trimester Following Iron Therapy to Achieve Non‐Anemic State by Delivery**
		**Rupsa C. Boelig^1^**; Mrutyunjaya Bellad^2^; Manjunath Somannavar^2^; Simal Thind^3^; Michael Georgieff^4^; Sudhir Bhandari^5^; Sudhir Mehta^5^; Seema Mehta^5^; Dharmesh Kumar Sharm^5^; Yogesh Kumar^2^; Umesh Charantimath^2^; Amaresh Patil^2^; Ashalata Mallapur^6^; Umesh Ramadurg^6^; Radha Sangavi^7^; Praveen Patil^7^; Subarana Roy^8^; Phaniraj Vastrad^8^; Chander Shekhar^3^; Benjamin Leiby^3^; Rebecca Hartmann^3^; Richard Derman^3^ ^1^Sidney Kimmel Medical College, Thomas Jefferson University, Philadelphia, PA; ^2^KLE Academy of Higher Education and Research J N Medical College, Belagavi, Karnataka; ^3^Thomas Jefferson University, Philadelphia, PA; ^4^University of Minnesota, Minneapolis, MN; ^5^Sawai Man Singh Medical College, Jaipur, Rajasthan; ^6^S Nijalingappa Medical and HSK Hospital and Research Center, Bagalkote, Karnataka; ^7^Raichur Institute of Medical Sciences, Raichur, Karnataka; ^8^Model Rural Health Research Unit, Sirwar, Karnataka
2:45 PM	16	**Contemporary Patterns and Outcomes of Antidepressant Discontinuation in Pregnancy**
		**Kelly B. Zafman^1^**; Yifan Zhu^2^; Sara Kornfield^3^; Aaron Smith‐McLallen^2^; Sindhu K. Srinivas^4^ ^1^University of Pennsylvania, Philadelphia, PA; ^2^Independence Blue Cross, Philadelphia, PA; ^3^Hospital of the University of Pennsylvania, Hospital of the University of Pennsylvania, PA; ^4^Department of Obstetrics and Gynecology, Perelman School of Medicine, Maternal and Child Health Research Center, Philadelphia, PA

**Table:** 

Wednesday, February 11, 2026 1:00 PM – 3:30 PM		
Time	Abstract	Oral Concurrent Session 1 – Medical and Surgical Complications of Pregnancy, continued
3:00 PM	17	**Engagement Drives Detection: Rethinking Postpartum Behavioral Health Screening**
		**Miriam J. Alvarez^1^**; Anna Madden‐Rusnak^1^; Colby Brown^1^; Alejandro Guevara^2^; Vaishnavi V. Kankate^1^; Lorie M. Harper^1^; Nandini Raghuraman^3^; Erin Richardson^4^; Jeffrey Newport^5^; George A. A. Macones^1^; Alison G. G. Cahill^6^ ^1^Department of Women's Health, Dell Medical School at the University of Texas at Austin, Austin, TX; ^2^Texas Tech University Health Sciences Center Paul L. Foster School of Medicine, El Paso, TX; ^3^Washington University School of Medicine, Washington University School of Medicine, MO; ^4^Women's Reproductive Mental Health, Dell Medical School at the University of Texas at Austin, Austin, TX; ^5^Women's Reproductive Mental Health, Dell Medical School at the University of Texas at Austin, Austin, TX; ^6^Department of Women's Health, Dell Medical School at the University of Texas at Austin, Department of Women's Health, Dell Medical School at the University of Texas at Austin, TX
3:15 PM	18	**Placental Immune Cell and Oxygenation Profiles in Opioid‐Exposed Pregnancies: A Longitudinal MRI Study**
		**Jeannie C. Kelly^1^**; Zezhen Xiang^1^; Sam A. S. Williams^1^; Melissa Mills^2^; Emily Diveley, BSN^1^; Nandini Raghuraman^1^; Peinan Zhao^1^; Nardhy Gomez‐Lopez^1^; Antonina I. Frolova^3^; Yong Wang^1^ ^1^Washington University School of Medicine, Washington University School of Medicine, MO; ^2^Washington University School of Medicine, Saint Louis, MO; ^3^Washington University School of Medicine, St. Louis, MO

**Table:** 

Wednesday, February 11, 2026 1:00 PM – 3:30 PM		
Time	Abstract	Oral Concurrent Session 2 – Diabetes
1:00 PM	19	**Predictive Ability of Early Pregnancy Glycemic Markers for Gestational Diabetes and Large for Gestational Age**
		**Camille E. Powe^1^**; Margaret Banker^2^; Patrick M. Catalano^3^; Francesca Facco^4^; William A. Grobman^5^; Erin S. LeBlanc^6^; William L. Lowe^7^; Audrey Merriam^8^; Mirella Mourad^9^; Caryn E.S. Oshiro^10^; Uma M. Reddy^11^; Dwight J. Rouse^12^; Jennifer Sherr^13^; Alexandra C. Spadola^14^; Kimberly K. Vesco^6^; Erika F. Werner^14^; Lynn M. Yee^15^; Noelia Zork^11^; Denise Scholtens^15^; Maisa Feghali^16^ ^1^Massachusetts General Hospital, Boston, MA; ^2^Northwestern Feinberg School of Medicine, Chicago, IL; ^3^MGH REU, Boston, MA; ^4^UPMC, UPMC, PA; ^5^Warren Alpert Medical School of Brown University, Providence, RI; ^6^Kaiser Permanente Center for Health Research, Portland, OR; ^7^Northwestern University, Northwestern University, IL; ^8^Yale School of Medicine, Yale School of Medicine, CT; ^9^Division of Maternal‐Fetal Medicine, Department of Obstetrics and Gynecology, Columbia University Irving Medical Center, New York, NY; ^10^Kaiser Permanente Hawaii, Center for Integrated Health Care Research, Honolulu, HI; ^11^Columbia University Irving Medical Center, New York, NY; ^12^Women & Infants Hospital of Rhode Island/Warren Alpert Medical School of Brown University, Providence, RI; ^13^Yale School of Medicine, New Haven, CT; ^14^Tufts University School of Medicine, Boston, MA; ^15^Northwestern University Feinberg School of Medicine, Chicago, IL; ^16^Magee Women's Research Institute, University of Pittsburgh, Pittsburgh, PA
1:15 PM	20	**A RCT of Patient‐Led Insulin Titration for Glycemic Management With Gestational Diabetes Mellitus**
		**Xiao‐Yu Wang^1^**; William A. Grobman^2^; Jiqiang Wu^1^; Rita Suzawa^1^; Jenna Kralik^1^; Taryn L. Summerfield^1^; Shaylyn Vickers^1^; Brenda Widmayer^1^; Melissa Rainier^1^; Julie Somppi^1^; Lisa Buccilla^1^; Bridget Iadicicco^1^; Elizabeth O. Buschur^1^; Steven Gabbe^1^; Mark B. Landon^3^; Kartik K. Venkatesh^3^ ^1^The Ohio State University Wexner Medical Center, Columbus, OH; ^2^Warren Alpert Medical School of Brown University, Providence, RI; ^3^The Ohio State University Wexner Medical Center, The Ohio State University Wexner Medical Center, OH
1:30 PM	21	**Disposition Index During Pregnancy as a Predictor of Insulin Requirement in Women With Gestational Diabetes**
		**Sohei Hamazaki**; Hiroshi Yamashita; Ichiro Yasuhi NHO Nagasaki Medical Center, Omura, Nagasaki
1:45 PM	22	**Are Hofbauer Cells Inflammatory in Placentas From Pregnancies Complicated by Gestational Diabetes Mellitus?**
		**Morgan E. Wasickanin^1^**; Rachel Watkin^2^; Joshua Monson^2^; Sarah Ligon^2^; Samantha Ocampo^3^; Hillary Kinsman^2^; Jennifer Damicis^2^; Katherine Free^2^; Lydia Bettridge^2^; Emily D. Sheikh^2^; Robert Walton^2^; Peter Napolitano^4^; Nicholas Ieronimakis^2^ ^1^Madigan Army Medical Center, Fort Bragg, NC; ^2^Madigan Army Medical Center, Tacoma, WA; ^3^Oregon Health & Science University, Portland, OR; ^4^University of Washington, Seattle, WA
2:00 PM	23	**Intrapartum Glucose Monitoring of A2GDM: A Randomized Control Trial**
		**Thomas Owens^1^**; Erica Glaser^2^; Johanna A. Suskin^2^; Leonardo Antelo^2^; Guillaume Stoffels^3^; Mia Heiligenstein^4^; Xiteng Yan^2^; Zainab Al‐Ibraheemi^5^; Lois Brustman^2^ ^1^Icahn School of Medicine at Mount Sinai, Atlanta, GA; ^2^Icahn School of Medicine, Mount Sinai West, New York, NY; ^3^Icahn School of Medicine at Mount Sinai, New York, NY; ^4^Icahn School of Medicine, Mount Sinai West, New York City, NY; ^5^Mount Sinai West, New York City, NY
2:15 PM	24	**Choline Supplementation and Placenta Gene Expression in GDM Pregnancies**
		**Youstina Soliman^1^**; Isabelle Hanson^2^; Jeanette Hausser^2^; Xinyin Jiang^1^; Elizabeth A. Larson^3^; Rodney A. McLaren^1^; Itamar Futterman^4^ ^1^Maimonides Medical Center, Brooklyn, NY; ^2^Department of Health and Nutrition Sciences, Brooklyn College, Brooklyn, NY; ^3^Division of Nutritional Sciences, Cornell University, Ithaca, NY; ^4^Maimonides Medical Center, Maimonides Health, NY
2:30 PM	25	**AI in Action: Evaluating use of ChatGPT for Insulin Management in Gestational Diabetes Mellitus**
		**Lauren M. Murphy**; Amanda A. Allshouse; Lauren H. Theilen; Amy Sullivan; Michelle P. Debbink; Ware Branch University of Utah, Salt Lake City, UT
2:45 PM	26	**Investigating Continuous Glucose Monitoring (CGM) Metrics for Gestational Diabetes to Optimize Neonatal Health**
		**Amy M. Valent^1^**; Celeste Durnwald^2^; Katrina Ramsey^1^; Emily Fay^3^; Nicole M. Ehrhardt^3^
		^1^Oregon Health & Science University, Portland, OR; ^2^Perelman School of Medicine at the University of Pennsylvania, Philadelphia, PA; ^3^University of Washington, Seattle, WA
3:00 PM	27	**Timing of Metformin Initiation for Type 2 Diabetes in Pregnancy and Perinatal Outcomes**
		**Claire E. Jensen^1^**; Kim Boggess^2^; Kjersti M. Aagaard^3^ ^1^University of North Carolina at Chapel Hill, Durham, NC; ^2^University of North Carolina at Chapel Hill, Chapel Hill, NC; ^3^HCA, Houston, TX
3:15 PM	28	**Risk of Gestational Diabetes by Aerobic and Anaerobic Exercise During the First and Second Trimesters**
		**Takaki Tanamoto^1^**; Grace Spencer^2^; Tetsuya Kawakita^3^ ^1^Teine Keijinkai Hospital, Sapporo City, Hokkaido; ^2^Eastern Virginia Medical School at Old Dominion University, EVMS OBGYN, Virginia Health Sciences at Old Dominion University, VA; ^3^Eastern Virginia Medical School Department of Obstetrics and Gynecology, Macon & Joan Brock Virginia Health Sciences at Old Dominion University, Norfolk, VA

**Table:** 

Wednesday, February 11, 2026 1:00 PM – 3:00 PM		
Time	Abstract	Oral Concurrent Session 3 – Fetus and Fetal Intervention
1:00 PM	29	**Childhood Obesity and Other Outcomes in Offspring Exposed to Adjunctive Azithromycin From the C/SOAP Trial**
		**Akila Subramaniam^1^**; Justin M. Leach^1^; Jeff M. Szychowski^2^; Alan T. Tita^1^ On behalf of the C/SOAP Childhood Follow‐Up Study Consortium ^1^Center for Research in Women's Health, University of Alabama at Birmingham, Birmingham, AL; ^2^Department of Biostatistics, University of Alabama at Birmingham, Birmingham, AL
1:15 PM	30	**Prenatal Surgical‐to‐Delivery Interval and 30‐Month Neurodevelopment: A Secondary Analysis of the MOMS Cohort**
		**Nikan Zargarzadeh^1^**; Claudio V. Schenone^2^; Eyal Krispin^2^; Jonathan Castillo^3^; Heidi Castillo^3^; Alireza A. Shamshirsaz^4^; Ashish Premkumar^5^ ^1^Boston Children's Hospital, Boston, MA; ^2^Boston Children's Hospital, Harvard Medical School, Boston, MA; ^3^UNMC Division of Developmental Medicine, Omaha, NE; ^4^Boston Children's Hospital, Brigham and Women's Hospital, Beth Israel Deaconess Medical Center, Harvard Medical School, Boston, MA; ^5^University of Chicago, Chicago, IL
1:30 PM	31	**AI‐Powered Fetal Movement Detection via Smartphones With the Potential to Reduce Risk of Late Stillbirth**
		**Anna Madden‐Rusnak^1^**; Kenneth J. Moise, Jr.^1^; Kelly P. Gaither^2^; Kathy J. Lowry^1^; Emily Hutson^1^; Danielle Bruns^1^; Reinaldo Valero^1^ ^1^Department of Women's Health, Dell Medical School at the University of Texas at Austin, Austin, TX; ^2^Texas Advanced Computing Center, Austin, TX
1:45 PM	32	**Antenatal Risk Factors for Neonatal Hypoxic‐Ischaemic Encephalopathy: A Prospective National Population‐Based Case‐Control Study**
		**Pierre Delorme^1^**; Rayan Hamadmad^2^; Loïc Sentilhes^3^; Hugo Madar^3^; Isabelle Guellec^4^; Antoine Villotitch^5^; Camille Le Ray^6^; Thierry Debillon^7^; Anne Ego^5^; Gilles Kayem^8^ ^1^Sorbonne Université/Department of Obstetrics and Gynecology, Trousseau Hospital, AP‐HP, sorbonne université, Ile‐de‐France; ^2^Sorbonne Université, sorbonne universite, Ile‐de‐France; ^3^Bordeaux University Hospital, Bordeaux Universitary Hospital, Aquitaine; ^4^CHU de Nice ‐ Hôpital l'Archet, CHU DE NICE HOPITAL DE LARCHET, Provence‐Alpes‐Cote d'Azur; ^5^Univ. Grenoble Alpes, CNRS, Public Health Department CHU Grenoble Alpes, Grenoble, Univ. Grenoble Alpes, CNRS, Public Health Department CHU Grenoble Alpes, Grenoble, Auvergne; ^6^Department of Obstetrics and Gynecology, Port‐Royal Maternity Hospital, AP‐HP, Cochin Hospital, FHU PREMA, Paris, France, Paris, Ile‐de‐France; ^7^Neonatal Intensive Care Unit, Grenoble Alpes University Hospital, Grenoble, France, Neonatal Intensive Care Unit, Grenoble Alpes University Hospital, Grenoble, France, Auvergne; ^8^Trousseau Maternity, Paris, Ile‐de‐France
2:00 PM	33	**Fetal Hematologic Profile in Monochorionic Twins With Anemia‐Polycythemia Sequence Undergoing Serial Transfusion and Partial Exchange**
		**Alexandra D. Forrest^1^**; Jena L. Miller^2^; Michelle Kush^3^; Mara Rosner^2^; Ahmet A. Baschat^4^ ^1^Johns Hopkins University, Baltimore, MD; ^2^Johns Hopkins Center for Fetal Therapy, Johns Hopkins University/Baltimore, MD; ^3^Johns Hopkins Center for Fetal Therapy, Johns Hopkins University/BAltimore, MD; ^4^Johns Hopkins University, Johns Hopkins University, MD
2:15 PM	34	**Perioperative Middle Cerebral Artery Flow Changes in Twin‐Twin Transfusion Syndrome and 2‐Year Neurodevelopmental Outcome**
		**Ramen H. Chmait^1^**; Lisa M. Korst^2^; Arlyn S. Llanes^1^; Moshe Fridman^3^; Adam Beasley^4^; Douglas L. Vanderbilt^4^ ^1^Keck School of Medicine of USC, University of Southern California, Keck School of Medicine of USC, University Of Southern California, CA; ^2^Childbirth Research Associates, North Hollywood, CA; ^3^AMF Consulting, Los Angeles, CA; ^4^Children's Hospital Los Angeles, Los Angeles, CA

**Table:** 

Wednesday, February 11, 2026 1:00 PM – 3:00 PM		
Time	Abstract	Oral Concurrent Session 3 – Fetus and Fetal Intervention, continued
2:30 PM	35	**Postoperative Fetal Imaging is Predictive of Need for CSF Diversion After Fetal Spina Bifida Repair**
		**Katherine H. Bligard^1^**; Jesse Vrecenak^1^; Jennifer M. Strahle^2^; Sasidhar Karuparti^1^; Jagruti Anadkat^1^; Nandini Raghuraman^3^; Jeannie C. Kelly^3^; Sherri Jackson^3^; Bree A. Goodman^2^ ^1^Washington University School of Medicine, Saint Louis, MO; ^2^Washington University School of Medicine, St. Louis, MO; ^3^Washington University School of Medicine, Washington University School of Medicine, MO
2:45 PM	36	**Two Year Outcomes in Premature Infants Randomized to Umbilical Cord Milking Versus Delayed Cord Clamping**
		**Anup Katheria^1^**; Jeff M. Szychowski^2^; Nicole Wilson^3^; Kathy Arnell^4^; Yvonne Vaucher^4^ On behalf of the Premod2 Study Group ^1^Sharp Mary Birch Hospital for Women & Newborns, Sharp Mary Birch Hospital for Women & Newborns, CA; ^2^Department of Biostatistics, University of Alabama at Birmingham, Birmingham, AL; ^3^Department of Biostatistics, University of Alabama at Birmingham, Burmingham, AL; ^4^Sharp Mary Birch Hospital for Women & Newborns, San Diego, CA

**Table:** 

Thursday, February 12, 2026 8:00 AM – 10:00 AM		
Time	Abstract	Oral Plenary Session 2 – Fellows Plenary
8:00 AM	37	**Antepartum Magnesium Sulfate Before Delivery at 22–24 Weeks Gestation and Long‐Term Neurodevelopmental Outcomes**
		**Margaret R. Page^1^**; Barbara Do^2^; Namasivayam Ambalavanan^3^; Colm P. Travers^4^; Waldemar A. Carlo^5^; Samuel Gentle^3^; Amelia Schuyler^3^; Akila Subramaniam^1^; Ashley N. Battarbee^1^ On behalf of the Eunice Kennedy Shriver NICHD Neonatal Research Network ^1^Center for Research in Women's Health, University of Alabama at Birmingham, Birmingham, AL; ^2^RTI International, Research Triangle Park, NC; ^3^University of Alabama at Birmingham Heersink School of Medicine, Birmingham, AL; ^4^Department of Pediatrics, University of Alabama at Birmingham Heersink School of Medicine, Birmingham, AL; ^5^Department of Pediatrics, University of Alabama at Birmingham, Birmingham, AL
8:15 AM	38	**Repeat Type and Screen Testing in Antepartum Admissions: Where Is the Evidence?**
		**Kimen S. Balhotra^1^**; Aaron W. Roberts^2^; Farah H. Amro^1^; Baha M. Sibai^1^ ^1^Division of Maternal‐Fetal Medicine, Department of Obstetrics, Gynecology and Reproductive Sciences, McGovern Medical School at UTHealth Houston, Houston, TX; ^2^Department of Obstetrics, Gynecology and Reproductive Sciences, McGovern Medical School at UTHealth Houston, Houston, TX
8:30 AM	39	**AI Based Ultrasound Screening for Early, Accurate Identification of Placenta Accreta Spectrum**
		**Alexandra L. Hammerquist^1^**; Sanmay Sarada^2^; Yamely H. Mendez^1^; Christina C. Reed^1^; Enrico R. Barrozo^3^; Jessian L. Munoz^4^; Wesley Lee^2^; Amir A. Shamshirsaz^5^; Michael A. Belfort^1^; Michael D. Jochum, Jr.^1^; Hendrik A. Lombaard^1^ ^1^Baylor College of Medicine, Houston, TX; ^2^Baylor College of Medicine, Baylor College of Medicine, TX; ^3^Baylor College of Medicine and Texas Children's Hospital, Houston, TX; ^4^Texas Children's Hospital and Baylor College of Medicine, Houston, TX; ^5^Division of Maternal‐Fetal Medicine, Department of Obstetrics and Gynecology, Baylor College of Medicine, Houston, TX

**Table:** 

Thursday, February 12, 2026 8:00 AM – 10:00 AM		
Time	Abstract	Oral Plenary Session 2 – Fellows Plenary, continued
8:45 AM	40	**Intraplacental Gene Transfer of IGF‐1 Corrects Preeclampsia in BPH/5 Mouse**
		**Kristen L. Moriarty^1^**; Sanjukta Majumder^2^; Jennifer Sones^3^; Timothy Crombleholme^4^ ^1^UConn Health, Farmington, CT; ^2^Connecticut Children's Medical Center, Farmington, CT; ^3^University of Colorado, Fort Collins, CO; ^4^Connecticut Children's Medical Center, Hartford, CT
9:00 AM	41	**Dermabond Prineo Versus Subcuticular Suture for Cesarean Delivery Skin Closure: A Randomized‐Controlled Trial**
		**Kristine Brown^1^**; Mariam Ayyash^1^; Hooman A. Azad^2^; Lielle Yellin^1^; Olivia F. Schulist^1^; Shai Bejerano^1^; Helen B. Gomez Slagle^1^; Said Saab^1^; Jean‐Ju Sheen^3^; Russell S. Miller^4^; Uma M. Reddy^1^; Lilly Liu^5^ ^1^Columbia University Irving Medical Center, New York, NY; ^2^Columbia University, Columbia University, NY; ^3^New York Presbyterian, Columbia University Irving Medical Center, Northvale, NJ; ^4^Division of Maternal‐Fetal Medicine, Department of Obstetrics and Gynecology, Columbia University Irving Medical Center, New York, NY; ^5^Albert Einstein School of Medicine/Montefiore Medical Center, New York, NY
9:15 AM	42	**DIP: An RCT of Diabetes Screening Immediately Postpartum After GDM**
		**Christine P. Field^1^**; William A. Grobman^2^; Devra Mast^3^; Jiqiang Wu^3^; Shaylyn Vickers^3^; Veronica Csizmadia^3^; Anna Bartholomew^3^; Anna Chen^3^; Vidya Mullangi^3^; Steven Gabbe^3^; Elizabeth O. Buschur^3^; Erika F. Werner^4^; Mark B. Landon^1^; Kartik K. Venkatesh^1^ ^1^The Ohio State University Wexner Medical Center, The Ohio State University Wexner Medical Center, OH; ^2^Warren Alpert Medical School of Brown University, Providence, RI; ^3^The Ohio State University Wexner Medical Center, Columbus, OH; ^4^Tufts University School of Medicine, Boston, MA
9:30 AM	43	**TNF‐α Inhibition Improves Blood Pressure and Inflammation in a Preeclampsia Rat Model**
		**Baylee Brown^1^**; Nathan Campbell^1^; Evangeline Deer^1^; Rachael Morris^1^; Ty Turner^1^; Alexandra Demesa^1^; Kennedy Cleveland^1^; Baoying Zheng^1^; Babbette LaMarca^2^ ^1^University of Mississippi Medical Center, Jackson, MS; ^2^The University of Mississippi Medical Center, Jackson, MS
9:45 AM	44	**A Randomized Control Trial of Interventions to Increase Prenatal Tdap Acceptance Among Non‐Birthing Partners**
		**Laurie B. Griffin^1^**; Natalie Passarelli^2^; Maxine Slater^3^; John Soehl^4^; Erica Hardy^3^; Melissa A. Clark^5^; Adam K. Lewkowitz^6^ ^1^University of Utah, Cottonwood Heights, UT; ^2^Beth Israel Deaconess Medical Center, Boston, MA; ^3^Alpert Medical School of Brown University/Women & Infants Hospital of Rhode Island, Providence, RI; ^4^University of Wisconsin‐Madison, Madison, WI; ^5^Brown University School of Public Health, Providence, RI; ^6^Women & Infants Hospital of Rhode Island/Warren Alpert Medical School of Brown University, Providence, RI

**Table:** 

Thursday, February 12, 2026 10:00 AM – 10:30 AM		
Time	Abstract	Late‐Breaking Abstract Presentations Session 2
**10:00 AM**	**LB03**	**Behavioral Health‐Enhanced Group Prenatal Care Prevents Postpartum Depression: The EleVATE Trial**
		**Ebony Carter** ^1^; Traci Johnson^2^; Kelly McKay‐Gist^3^; Richelle Smith^3^; Cheron Phillips^3^; Julia Muller^4^; Rachel Paul^5^; Jeannie C. Kelly^6^; Nandini Raghuraman^7^; Melissa Tepe^8^; Candice L. Woolfolk^6^; Shannon Lenze^9^ On behalf of Elevating Voices, Addressing depression, and Toxic Stress (EleVATE) through Group Prenatal Care Collaborative
		^1^University of North Carolina, Chapel Hill, NC; ^2^University of Missouri‐Kansas City, Kansas City, MO; ^3^St. Louis Integrated Health Network, St. Louis, MO; ^4^University of North Carolina, Chapel Hill, NC; ^5^Washington University School of Medicine, Washinton University in St. Louis School of Medicine, MO; ^6^Washington University School of Medicine, Washington University School of Medicine, MO; ^7^Washington University School of Medicine, St. Louis, MO; ^8^Affinia Healthcare, St. Louis, MO; ^9^Washington University in St. Louis, School of Medicine, Department of Psychiatry, St. Louis, MO
**10:15 AM**	**LB04**	**Pregnancy Antihypertensive Drugs: Which Agent Is Best? The Giant PANDA Randomised Controlled Trial**
		**Jenny E. Myers^1^**; Alisha Maher^2^; Catherine Moakes^2^; Lucy C. Chappell^3^ On behalf of the Giant PANDA Investigator Group
		^1^University of Manchester, Manchester, England; ^2^University of Birmingham, Birmingham, England; ^3^King's College London, London, England

**Table:** 

Thursday, February 12, 2026 1:00 PM – 3:15 PM		
Time	Abstract	Oral Concurrent Session 4 – Equirty, Public Health, and Public Policy
1:00 PM	45	**Impact of a Substance Use Screening Program on Racial Disparities in Urine Toxicology Testing**
		**Mariam Naqvi^1^**; Amin Tavakoli^2^; Camelita Thrift^3^; Allison Henry^4^; Jennifer Astasio^1^; Naomi H. Greene^3^; Sarah Kilpatrick^1^ ^1^Department of Obstetrics and Gynecology, Cedars‐Sinai Medical Center, Los Angeles, CA; ^2^Department of Obstetrics and Gynecology, Cedars‐Sinai Medical Center, Los Angeles, CA, CA; ^3^Department of Obstetrics & Gynecology, Cedars‐Sinai Medical Center, Los Angeles, CA; ^4^Department of Pediatrics, Cedars‐Sinai Medical Center, Los Angeles, CA
1:15 PM	46	**Experiences of Discrimination and Adverse Pregnancy Outcomes—A Secondary a Analysis of the EMBRACE Comparative‐Effectiveness Trial**
		**Daisy León‐Martínez^1^**; Deborah Karasek^2^; Brittany D. Chambers Butcher^3^; Cinthia Blat^4^; Venise Curry^5^; Patience A. Afulani^6^; Charles E. McCulloch^7^; Miriam Kuppermann^8^ ^1^Division of Maternal‐Fetal Medicine at the University of California San Francisco, University of California San Francisco, CA; ^2^Oregon Health & Science University‐Portland State University School of Public Health, Portland, OR; ^3^Department of Human Ecology, College of Agricultural and Environmental Sciences, University of California, Davis, Davis, CA; ^4^Department of Obstetrics, Gynecology and Reproductive Sciences, University of California, San Francisco, San Francisco, CA; ^5^Central Valley Health Policy Institute at California State University, Fresno, Fresno, CA; ^6^Department of Obstetrics, Gynecology and Reproductive Sciences, University of California, San Francisco, Universiy of California San Francisco, CA; ^7^University of California, San Francisco, University of California San Francisco, CA; ^8^Department of Obstetrics, Gynecology and Reproductive Sciences, University of California, San Francisco, University of California San Francisco, CA

**Table:** 

Thursday, February 12, 2026 1:00 PM – 3:15 PM		
Time	Abstract	Oral Concurrent Session 4 – Equirty, Public Health, and Public Policy, continued
1:30 PM	47	**Exploring Neighborhood‐Level Indicators of Social Disadvantage Associated With Prenatal Inflammation**
		**Sarah L. Kaplan^1^**; Samantha P. Gottlieb^2^; Frances Kincaid^3^; Estefania Espinosa^4^; Kennedi Williams^4^; Jiafeng Li^5^; Madeline R. Meirhaeghe^6^; Abigail L. Aron^7^; Mary Akel^5^; Leena B. Mithal^8^; Tonia Branche^5^; Stephanie A. Fisher^9^ ^1^Ann & Robert H. Lurie Children's Hospital of Chicago, Atlanta, GA; ^2^Ann & Robert H. Lurie Children's Hospital of Chicago, Lurie Children's Hospital, Chicago, IL, IL; ^3^Northwestern University, Northwestern University, IL; ^4^Northwestern University, Chicago, IL; ^5^Ann & Robert H. Lurie Children's Hospital of Chicago, Chicago, IL; ^6^Ann & Robert H. Lurie Children's Hospital of Chicago, LIncolnshire, IL; ^7^Ann & Robert H. Lurie Children's Hospital of Chicago, Ann & Robert H. Lurie Children's Hospital of Chicago, IL; ^8^Ann & Robert H. Lurie Children's Hospital; Northwestern University Feinberg School of Medicine, Chicago, IL; ^9^Northwestern University Feinberg School of Medicine, Chicago, IL
1:45 PM	48	**State Abortion Restrictions and Maternal Deaths in the United States: 2005–2023**
		**Marie C. Anderson^1^**; Hooman A. Azad^2^; Dana Goin^2^; Uma M. Reddy^3^; Mary E. D'Alton^4^; Erika E. Levi^5^; Lisa Nathan^3^ ^1^Columbia University, New York, NY; ^2^Columbia University, Columbia University, NY; ^3^Columbia University Irving Medical Center, New York, NY; ^4^Division of Maternal‐Fetal Medicine, Department of Obstetrics and Gynecology, Columbia University Irving Medical Center, New York, NY; ^5^Columbia University, Columbia Univeristy, NY
2:00 PM	49	**Association Between Trial of Labor and Vaginal Birth After Cesarean Odds and Hospital‐Level Racial Segregation**
		**Max Jordan Nguemeni Tiako^1^**; Chong Zhang^2^; Jaewhan Kim^2^; Adebayo Adesomo^3^; Michelle P. Debbink^4^ ^1^University of California, Los Angeles (UCLA), Los Angeles, CA; ^2^University of Utah, University of Utah, UT; ^3^Utah Health, Utah Health, UT; ^4^University of Utah, Salt Lake City, UT
2:15 PM	50	**Patient Navigation and Postpartum Patient‐Reported Outcomes Among Low‐Income Birthing People: A Randomized Controlled Trial**
		**Sydney L. Raucher^1^**; Brittney R. Williams^1^; Joseph M. Feinglass^1^; Ying Cheung^1^; Laura Diaz^1^; Ka'Derricka Davis^1^; Michelle A. Kominiarek^1^; William A. Grobman^2^; Lynn M. Yee^1^ ^1^Northwestern University Feinberg School of Medicine, Chicago, IL; ^2^Warren Alpert Medical School of Brown University, Providence, RI
2:30 PM	51	**Maternal Dietary Trajectories and Urinary Analyte Signatures Associate With Preterm Birth**
		Michael D. Jochum, Jr.^1^; Cynthia Shope^2^; Melissa A. Suter^3^; Kjersti M. Aagaard^4^; **Enrico R. Barrozo^3^** ^1^Baylor College of Medicine, Houston, TX; ^2^Baylor College of Medicine, Baylor College of Medicine, TX; ^3^Baylor College of Medicine and Texas Children's Hospital, Houston, TX; ^4^HCA, Houston, TX
2:45 PM	52	**Ultrasound Practice Benchmarks in Maternal Fetal Medicine Units in the United States**
		**Fadi Bsat^1^**; Suwan Mehra^2^ ^1^SMFM Practice Management Committee, West Springfield, MA; ^2^Advocate Children's Hospital, Advocate Childrens Hospital, IL
3:00 PM	53	**Promotion of RSV Immunization Through Multiple Efforts (PRIME): A Sequential Multiple Assignment Randomized Trial**
		**Jeannie C. Kelly^1^**; Abigail Arter^1^; Candice L. Woolfolk^1^; Elvin Geng^1^; Jason Newland^2^; Antonina I. Frolova^3^; Nandini Raghuraman^1^ ^1^Washington University School of Medicine, Washington University School of Medicine, MO; ^2^Nationwide Children's Hospital, Nationwide Children's Hospital, OH; ^3^Washington University School of Medicine, St. Louis, MO

**Table:** 

Thursday, February 12, 2026 1:00 PM – 3:30 PM		
Time	Abstract	Oral Concurrent Session 5 – Clinical Obstetrics
1:00 PM	54	**Adoption and Real‐World Effects of Azithromycin for Labored Cesarean Birth on Postpartum Infections**
		**Taylor S. Freret^1^**; Ethan Litman^2^; Mark A. Clapp^3^; Sarah E. Little^4^ ^1^Beth Israel Deaconess Medical Center, Brookline, MA; ^2^Beth Israel Deaconess Medical Center, Boston, MA; ^3^Department of Obstetrics and Gynecology, Mass General Brigham, Massachusetts General Hospital, MA; ^4^Beth Israel Deaconess Medical Center, Newton, MA
1:15 PM	55	**Intravenous Papaverine Prior to Dinoprostone for Labor Induction: A Randomized Double‐Blinded Placebo‐Controlled Trial**
		**Raneen Abu Shqara^1^**; Yara Bishara^2^; Nadir Ganem^3^; Inshirah Sgayer^2^; Miri Lavinsky^4^; Lior Lowenstein^3^; Maya Frank Wolf^3^ ^1^Galilee Medical Center, Nahariya, HaZafon; ^2^Galilee Medical Center, Nahariyya, HaZafon; ^3^Galilee Medical Center, Naharyia, HaZafon; ^4^Galilee Medical Center, Galilee Medical Center, HaZafon
1:30 PM	56	**Effect of Oral Calcium Carbonate on Uterine Contractility and Labor Outcomes: A Randomized Controlled Trial**
		**Tina M. Bui**; Michelle Nelson; Preeanka Mazumder; Rabia Rehman; Kristina A. Roloff; Guillermo Valenzuela Arrowhead Regional Medical Center, Colton, CA
1:45 PM	57	**Azithromycin Dosing in Preterm Premature Rupture of Membranes (PPROM): Population Pharmacokinetics and Dosing Regimen Optimization**
		**Rupsa C. Boelig^1^**; Kevin Lam^2^; Viren Soni^2^; Ankit Rochani^3^; Gagan Kaushal^4^; Amanda Roman^2^; Walter Kraft^2^ ^1^Sidney Kimmel Medical College, Thomas Jefferson University, Philadelphia, PA; ^2^Thomas Jefferson University, Philadelphia, PA; ^3^St. John Fisher University, Rochester, NY; ^4^Thomas Jefferson University, Thomas Jefferson University, PA
2:00 PM	58	**Non‐Inferiority Analysis of an FHR AI Algorithm to Clinician Interpretation**
		**Rohit Pardasani^1^**; Karen P. Becker^2^; Renee Vitullo^1^; Sara M. Harris^3^; Jaydev Kamani^4^; Jack Page^4^; Kritika Garg^4^; John Beard^1^ ^1^GE HealthCare, Waukesha, WI; ^2^GE HealthCare, waukesha, MD; ^3^GE HealthCare, Cape Coral, FL 33991‐3441, FL; ^4^GE HealthCare, waukesha, WI
2:15 PM	59	**Second Stage Cesarean Delivery: A Mixed‐Methods Exploration of Techniques and Uncertainty Among U.S. Clinicians**
		**Janayah McClellan^1^**; Erica Marion^1^; Sarah T. McMahon^1^; Melissa A. Clark^2^; Anna Palatnik^3^; Brock E. Polnaszek^1^ ^1^Medical College of Wisconsin, Milwaukee, WI; ^2^Brown University School of Public Health, Providence, RI; ^3^Medical College of Wisconsin, MILWAUKEE, WI
2:30 PM	60	**Membrane Sweeping Versus Double‐Balloon Catheter for Cervical Ripening: A Randomized Controlled Trial**
		Renana Izakson^1^; May Weinberg^2^; Michal Kovo^3^; Tal Biron‐Shental^4^; **Gal Cohen^4^** ^1^Meir Medical Center, meir medical center, HaMerkaz; ^2^Meir Medical Center, kfar saba, HaMerkaz; ^3^Shamir Medical Center, Beer Yaacov, HaMerkaz; ^4^Meir Medical Center, Kfar Saba, HaMerkaz

**Table:** 

Thursday, February 12, 2026 1:00 PM – 3:30 PM		
Time	Abstract	Oral Concurrent Session 5 – Clinical Obstetrics, continued
2:45 PM	61	**Factors Associated With a Restart of Oxytocin After Discontinuation in Active First Stage of Labor**
		Charles Garabedian^1^; Loïc Sentilhes^2^; Raoul Desbrière^3^; Paul Berveiller^4^; Diane Korb^5^; Charline Bertholdt^6^; Julie Carrara^7^; Norbert Winer^8^; Camille Le Ray^9^; **Aude Girault^9^** ^1^CHU Lille, CHU Lille, Department of obstetrics, Univ. Lille, ULR2694–METRICS, F59000 Lille, France, Nord‐Pas‐de‐Calais; ^2^Bordeaux University Hospital, Bordeaux Universitary Hospital, Aquitaine; ^3^Department of Obstetrics and Gynecology, Hôpital Saint Joseph, Marseille, France, Marseille, Midi‐Pyrenees; ^4^Department of Obstetrics and Gynecology, Centre Hospitalier Intercommunal de Poissy/ Saint‐Germain‐en‐Laye, Rue du Champ Gaillard, Poissy Cedex, France, Poissy, Ile‐de‐France; ^5^Department of Obstetrics and Gynecology, Robert Debré Hospital, AP‐HP, Paris, France, Paris, Ile‐de‐France; ^6^University of Lorraine, CHRU NANCY, Obstetrics and Gynecology Department, NANCY, France, Nancy, Lorraine; ^7^Department of Obstetrics and Gynecology, Antoine Béclère Hospital, AP‐HP, Paris, France, Clamart, Ile‐de‐France; ^8^Department of Obstetrics and Gynecology, University Hospital of Nantes, Nantes, Pays de la Loire; ^9^Department of Obstetrics and Gynecology, Port‐Royal Maternity Hospital, AP‐HP, Cochin Hospital, FHU PREMA, Paris, France, Paris, Ile‐de‐France
3:00 PM	62	**Prelabor Maternal Pushing Training Using Visual Biofeedback via Self‐Operated Home Ultrasound: A Randomized Controlled Study**
		**Natav Hendin^1^**; Amit Shachak^2^; Dorit Asher^3^; Eran Hadar^3^; Yael Benyamini^4^; Sharon Perlman^3^ ^1^Rabin Medical Center, Petah Tikva, HaMerkaz; ^2^Rabin medical center, Petach Tikva, HaMerkaz; ^3^Rabin Medical Center, Petach Tikva, HaMerkaz; ^4^Tel Aviv University, Tel Aviv, Tel Aviv
3:15 PM	63	**The Cumulative Impact of Category II Tracings Throughout Labor on Neonatal Lactate**
		**Nandini Raghuraman^1^**; Antonina I. Frolova^2^; Megan L. Lawlor^2^; Amanda C. Zofkie^2^; Roxane Rampersad^2^; Jeannie C. Kelly^1^; Steve Porter^3^; Susan Mann^4^; George A. A. Macones^5^; Alison G. G. Cahill^6^ ^1^Washington University School of Medicine, Washington University School of Medicine, MO; ^2^Washington University School of Medicine, St. Louis, MO; ^3^Case Western Reserve University, University Hospitals MacDonald Women's Hospital, Cleveland, OH; ^4^Beth Israel Deaconess Medical Center, Harvard Medical School, Boston, MA; ^5^Department of Women's Health, Dell Medical School at the University of Texas at Austin, Austin, TX; ^6^Department of Women's Health, Dell Medical School at the University of Texas at Austin, Department of Women's Health, Dell Medical School at the University of Texas at Austin, TX

**Table:** 

Thursday, February 12, 2026 1:00 PM – 3:30 PM		
Time	Abstract	Oral Concurrent Session 6 – Ultrasound and Genetrics
1:00 PM	64	**Rethinking CF Carrier Screening: Comprehensive CFTR Testing vs. Limited Variant Sets**
		**Mona M. Makhamreh^1^**; Erica Soster^2^; Jennifer Teicher^2^; April D. Adams^1^; Brandy Firman^3^; Virali Patel^4^; Huda B. Al‐Kouatly^5^ ^1^Baylor College of Medicine, Houston, TX; ^2^Labcorp, Westborough, MA; ^3^Thomas Jefferson University, Philadelphia, TX; ^4^Thomas Jefferson University, Philadelphia, PA; ^5^Division of Maternal‐Fetal Medicine, Department of Obstetrics and Gynecology, Sidney Kimmel Medical College at Thomas Jefferson University, Philadelphia, PA, USA, Philadelphia, PA
1:15 PM	65	**Development and Validation of a SNP‐Based cfDNA Test for Vanishing Twin Pregnancies: The VANISH Trial**
		**Lorraine Dugoff^1^**; Sunni L. Mumford^1^; Meredith Rochon^2^; Martin Chavez^3^; Katherine Kohari^4^; Jessica Scholl^5^; Jill Berkin^6^; Ashley S. Roman^7^; Jane Chueh^8^; Samuel Wolf^9^; Kristy Palomares^10^; K. Joseph Hurt^11^; Kelly Gorman^12^; Myra Wick^13^; Zeynep Alpay Savasan^14^; Lawrence D. Platt^15^; Marjorie Treadwell^16^; Abdulla Al‐Khan^17^; Matthew K. Hoffman^18^; Sara Rabin‐Havt^19^; Nicholas Sun^20^; Anastasia Butskova^20^; Wenbo Xu^20^; Vivienne Souter^20^ ^1^Perelman School of Medicine at the University of Pennsylvania, Philadelphia, PA; ^2^Lehigh Valley Health Network, Allentown, PA; ^3^NYU Grossman Long Island School of Medicine, Mineola, NY; ^4^Yale School of Medicine and Yale New Haven Hospital, New Haven, CT; ^5^Weill Cornell Medicine, New York, NY; ^6^Icahn School of Medicine at Mount Sinai, New York, NY; ^7^Division of Maternal‐Fetal Medicine, Department of Obstetrics and Gynecology, NYU Grossman School of Medicine, New York, NY; ^8^Stanford University, Stanford University, CA; ^9^Emerald Coast Obstetrics and Gynecology, Panama City, FL; ^10^St. Peter's University Hospital, New Brunswick, NJ; ^11^University of Colorado School of Medicine, Aurora, CO; ^12^University of Kansas School of Medicine, Kansas City, KS; ^13^Mayo Clinic, Rochester, MN; ^14^Corewell Health William Beaumont University Hospital, Royal Oak, MI; ^15^UCLA David Geffen School of Medicine and Cedars‐Sinai Medical Center, Los Angeles, CA; ^16^University of Michigan Hospital, University of Michigan, Ann Arbor, MI; ^17^Hackensack University Medical Center, Hackensack University Medical Center, NJ; ^18^Christiana Care Health System, Newark, DE; ^19^Montefiore Medical Center, Albert Einstein College of Medicine, Bronx, NY; ^20^Natera, Inc., Austin, TX
1:30 PM	66	**AI Estimation of Fetal Weight From Ultrasound Sweeps of the Gravid Abdomen**
		**Teeranan Pokaprakarn^1^**; Katelyn J. Rittenhouse^1^; Juan C. Prieto^1^; Margaret Kasaro^2^; Srihari Chari^1^; Yuri Sebastião^1^; Elizabeth M. M. Stringer^1^; Nicole Davis^1^; Mark Klose^1^; Rebecca Ritter^1^; M. Bridget Spelke^1^; Bellington Vwalika^3^; Stephen Cole^1^; Michael R. Kosorok^1^; Jeffrey S.A. Stringer^1^ ^1^University of North Carolina at Chapel Hill, Chapel Hill, NC; ^2^UNC Global Projects Zambia, Chapel Hill, NC; ^3^University of Zambia School of Medicine, Chapel Hill, NC
1:45 PM	67	**Regions of Homozygosity on Prenatal Chromosomal Microarray and Adverse Pregnancy Outcomes**
		**Victoria Ly^1^**; Nicola F. Tavella^2^; Jo Hsuan Lee^3^; Grace M. DiGiovanni^4^; Nina G. Faynshtayn^4^; Barbara Pereira Vera^4^; Shrinkhala Kaphle^5^; Huda B. Al‐Kouatly^6^; Angela T. Bianco^4^; Laura C. Gilroy^7^ ^1^Division of Maternal‐Fetal Medicine, Icahn School of Medicine at Mount Sinai, New York City, NY; ^2^Division of Maternal‐Fetal Medicine, Icahn School of Medicine, Icahn School of Medicine at Mount Sinai Hospital, New York, NY; ^3^Icahn School of Medicine at Mount Sinai, Icahn School of Medicine at Mount Sinai, NY; ^4^Icahn School of Medicine at Mount Sinai, New York, NY; ^5^Icahn School of Medicine at Mount Sinai, New York CIty, NY; ^6^Division of Maternal‐Fetal Medicine, Department of Obstetrics and Gynecology, Sidney Kimmel Medical College at Thomas Jefferson University, Philadelphia, PA, USA, Philadelphia, PA; ^7^Mount Sinai Hospital, Mount Sinai Hospital, NY

**Table:** 

Thursday, February 12, 2026 1:00 PM – 3:30 PM		
Time	Abstract	Oral Concurrent Session 6 – Ultrasound and Genetrics, continued
2:00 PM	68	**Validation of an AI Software for Major CHD Screening in a Prenatal Diagnostic Center**
		**Jennifer Lam‐Rachlin^1^**; Nathan S. Fox^2^; Clementine Roth^3^; Simi Gupta^2^; Shari Gelber^1^; Jessica Spiegelman^2^; Steven R. Inglis^2^; Celia Muoser^2^; Xiangna Tang^2^; Luciana Vieira^2^; Samantha Do^2^; Andrei Rebarber^4^ ^1^Icahn School of Medicine at Mount Sinai, Icahn School of Medicine at Mount Sinai, NY; ^2^Icahn School of Medicine, Mount Sinai West, New York, NY; ^3^Carnegie Imaging for Women PLLC, New York, NY; ^4^Icahn School of Medicine, Mount Sinai West, Mount Sinai West, NY
2:15 PM	69	**Sonographic Quantitative Vascularization of the Anterior Fornix/Cervix for Prediction Of Outcomes in Placenta Accreta Spectrum**
		**Elias Kassir^1^**; Edgar A. Hernandez‐Andrade^2^; Donatella Gerulewicz^2^; Sarah T. Mehl^2^; Farah H. Amro^2^; Eleazar E. Soto^1^; Ramesha Papanna^1^; Sean C. Blackwell^2^; Baha M. Sibai^2^ ^1^University of Texas Health Science Center in Houston, McGovern Medical School, Houston, TX; ^2^Division of Maternal‐Fetal Medicine, Department of Obstetrics, Gynecology and Reproductive Sciences, McGovern Medical School at UTHealth Houston, Houston, TX
2:30 PM	70	**Unsupervised Machine Learning of Fetal Myocardial Performance Index Trajectories Identifies FGR and SGA Patterns**
		**Rebecca Horgan^1^**; Erkan Kalafat^2^; Elena Sinkovskaya^1^; Alfred Abuhamad^1^; George R. Saade^1^ ^1^Eastern Virginia Medical School at Old Dominion University, Norfolk, VA; ^2^Koc University Hospital, Istanbul, Koc University Hospital, Istanbul, Istanbul
2:45 PM	71	**pcfDNA Screening With Fetal Fraction Amplification Between 8‐10 Weeks: Laboratory Experience With >28k Samples**
		**Ronit Lebor^1^**; D. Claire Miller^2^; Jack Roach^2^; Jhett Bordwell^2^; Seyedmehdi Shojaee^2^; Catherine Wicklund^2^; Summer Pierson^2^; Katie Johansen Taber^2^; Dale Muzzey^2^ ^1^Myriad Genetics, Inc., Philadelphia, PA; ^2^Myriad Genetics, Inc., Salt Lake City, UT
3:00 PM	72	**Incremental Diagnostic Yield of Genome Sequencing for Nonimmune Hydrops Fetalis Spectrum Disorders**
		**Rozlyn C.T Boutin^1^**; Billie R. Lianoglou^1^; Nuriye Sahin‐Hodoglugil^1^; Katie Tick^1^; Carmen M.A. Santoli^2^; Jessica Van Ziffle^1^; Patrick Devine^1^; Ugur Hodoglugil^3^; Pierre Martin^1^; Mary E. Norton^1^; Teresa N. Sparks^1^ ^1^University of California, San Francisco, San Francisco, CA; ^2^University of California, San Francisco, University of California, San Francisco, CA; ^3^University of California, San Francisco, San Fraincisco, CA
3:15 PM	73	**Cost‐Effectiveness of First Trimester Ultrasound After Low‐Risk Non‐Invasive Prenatal Testing**
		**Courtney Hargreaves^1^**; Mohak Mhatre^2^ ^1^Tufts University School of Medicine, Tufts University School of Medicine, MA; ^2^Tufts University School of Medicine, Boston, MA

**Table:** 

Friday, February 13, 2026 8:00 AM – 10:00 AM		
Time	Abstract	Oral Concurrent Session 7 – Basic and Translational Science
8:00 AM	74	**Proteomic Profile of Placenta Accreta Spectrum Highlighting Dysregulation and Paracrine Effects Adjacent to Cesarean Scar**
		Jonathan L. Hecht^1^; Anna M. Modest^1^; Uma S. Deshmukh^1^; Saira L. Salahuddin^1^; Simon T. Dillon^1^; Yalda Afshar^2^; S Ananth Karumanchi^3^; Towia A. Libermann^1^; **Scott A. Shainker^4^** ^1^Beth Israel Deaconess Medical Center, Boston, MA; ^2^David Geffen School of Medicine at University of California, Los Angeles (UCLA), Los Angeles, CA; ^3^Cedars‐Sinai Medical Center, eCedars‐Sinai Medical Center,, CA; ^4^Department of Obstetrics and Gynecology, Beth Israel Deaconess Medical Center, Harvard Medical School, Boston, MA
8:15 AM	75	**Unraveling the Pathogenesis of Common Pregnancy Complications: An Atlas of 1.2 Million Placental Cells**
		Paola A. Lopez‐Zapana^1^; Rachelly Normand^2^; Daehee Han^3^; Courtney Ambrose^4^; Roya Best^5^; Zhaojing Liu^1^; Elizabeth Tuttle^5^; Christopher Stueber^5^; Olyvia J. Jasset^1^; Laura Ibanez‐Pintor^1^; Lydia L. Shook^6^; Douglas A. Lauffenburger^7^; Alexandra‐Chloé Villani^8^; **Andrea G. Edlow^9^** ^1^Vincent Center for Reproductive Biology, Massachusetts General Hospital, Boston, MA; ^2^Massachusetts General Hospital, Massachusetts General Hospital, MA; ^3^Vincent Center for Reproductive Biology, Massachusetts General Hospital; Department of Obstetrics and Gynecology, MassGeneral Brigham; Division of Maternal‐Fetal Medicine; Harvard Medical School, Boston, MA; ^4^Center for Immunology and Inflammatory Diseases, Department of Medicine and Krantz Family Center for Cancer Research, Massachusetts General Hospital, Boston, MA; ^5^Massachusetts General Hospital, Boston, MA; ^6^Department of Obstetrics and Gynecology, MassGeneral Brigham; Division of Maternal‐Fetal Medicine; Harvard Medical School, Massachusetts General Hospital/Boston, MA; ^7^Department of Biological Engineering, Massachusetts Institute of Technology, Cambridge, MA; ^8^Center for Immunology and Inflammatory Diseases, Krantz Family Center for Cancer Research, Massachusetts General Hospital, Boston, MA; ^9^Department of Obstetrics and Gynecology, MassGeneral Brigham; Division of Maternal‐Fetal Medicine; Harvard Medical School, Boston, MA
8:30 AM	76	**Uterine Collagen Gradients for Placenta Accreta Prevention Utilizing Second Harmonic Imaging Microscopy**
		**Sohum C. Shah^1^**; Haley Marks^2^; Guadalupe Martinez^3^; Jakub Staniczek^4^; Alex Kot^2^; Roya Bagheri^5^; Deborah Krakow^6^; Yalda Afshar^6^ ^1^Department of Obstetrics and Gynecology, David Geffen School of Medicine at University of California, Los Angeles (UCLA), UCLA, CA; ^2^University of California, Los Angeles (UCLA), UCLA, CA; ^3^University of California, Los Angeles (UCLA), Los Angeles, CA; ^4^Śląski Uniwersytet Medyczny w Katowicach, Katowice, Slaskie; ^5^University of California, Los Angeles (UCLA), UCLA Health, CA; ^6^David Geffen School of Medicine at University of California, Los Angeles (UCLA), Los Angeles, CA
8:45 AM	77	**Urinary Lipidomic Profiling Associated With PM2.5 Exposure During Pregnancy**
		**Sunwha Park^1^**; Minki Shim^2^; Young‐Ah You^3^; Young Min Hur^4^; Yoon‐Young Go^3^; Mi Hye Park^4^; Young‐Han Kim^5^; Sung Hun Na^6^; Geum Joon Cho^7^; Jin‐Gon Bae^8^; Soo‐Jeong Lee^9^; Dong‐Kyu Lee^2^; Young Ju Kim^4^ ^1^Ewha Womans University Hospital, Ewha Womans University Hospital/Seoul, Seoul‐t'ukpyolsi; ^2^College of Pharmacy, Chung‐Ang University, Seoul, Seoul‐t'ukpyolsi; ^3^Ewha Medical Research Institute, Ewha Womans University, Seoul, Seoul‐t'ukpyolsi; ^4^Ewha Womans University Hospital, Seoul, Seoul‐t'ukpyolsi; ^5^Department of Obstetrics and Gynecology, Yonsei University College of Medicine, Department of Obstetrics and Gynecology, Yonsei University College of Medicine, Seoul‐t'ukpyolsi; ^6^Department of Obstetrics and Gynecology, Kangwon National University, School of Medicine, Department of Obstetrics and Gynecology, Kangwon National University, School of Medicine, Kangwon‐do; ^7^College of Medicine, Korea University, Seoul, Seoul‐t'ukpyolsi; ^8^Department of Obstetrics and Gynecology, Keimyung University, School of Medicine, Department of Obstetrics and Gynecology, Keimyung University, School of Medicine, Taegu‐Jikhalsi; ^9^University of Ulsan College of Medicine, Ulsan, Ulsan‐Gwangyoksi

**Table:** 

Friday, February 13, 2026 8:00 AM – 10:00 AM		
Time	Abstract	Oral Concurrent Session 7 – Basic and Translational Science, continued
9:00 AM	78	**Contribution of Antibody Subtypes to Breastmilk Immunity Against COVID‐19 in Disease and Vaccine Exposed Pregnancies**
		**Mymy Nguyen^1^**; Elke S. Bergmann‐Leitner^2^; Julie A. Jones^1^; Gregory Gromowski^2^; Zubair H. Aghai^3^; Rupsa C. Boelig^4^ ^1^Thomas Jefferson University Hospital, Philadelphia, PA; ^2^Walter Reed Army Institute of Research, Silver Spring, MD; ^3^Nemours Children's Health, Philadelphia, PA; ^4^Sidney Kimmel Medical College, Thomas Jefferson University, Philadelphia, PA
9:15 AM	79	**Efficacy of Novel Peptide B7‐33 in Attenuating Preeclampsia Symptoms: Preclinical Study in Rat Models**
		**Rachel M. Terry^1^**; Ahmed F. Pantho^2^; Michelle N. Harris^1^; Kelsey R. Kelso^1^; Niraj Vora^3^; Lorena M. Amaral^4^; Diana Villazana‐Kretzer^3^; Thomas Kuehl^2^; Babbette LaMarca^4^; Mohammad N. Uddin^5^ ^1^Baylor Scott & White Health ‐ Baylor College of Medicine, Temple, TX; ^2^Orion Institute for Translational Medicine, Georgetown, TX; ^3^Baylor Scott & White Medical Center, Temple, TX; ^4^The University of Mississippi Medical Center, Jackson, MS; ^5^Texas A&M Health University College of Medicine, Temple, TX
9:30 AM	80	**Prospective Evaluation of Platelet Hyperreactivity and Risk of Ischemic Placental Disease**
		**Christina A. Penfield^1^**; Andre A. Robinson^1^; Anais Hausvater^2^; Ariel Schapp^3^; Elliot Luttrell‐Williams^2^; Madison S. House^4^; Yuhe Xia^5^; Valeryia Avtushka^1^; Lauren Kennedy^2^; Rafid Quadir^2^; Justin S. Brandt^6^; Gwendolyn P. Quinn^7^; Ashley S. Roman^6^; Dana R. Gossett^7^; Jeffrey S. Berger^2^ ^1^Division of Maternal‐Fetal Medicine, Department of Obstetrics and Gynecology, NYU Grossman School of Medicine, New York City, NY; ^2^Leon H. Charney Division of Cardiology, Department of Medicine, NYU Grossman School of Medicine, New York City, NY; ^3^Division of Hematology Department of Medicine, NYU Grossman School of Medicine, New York City, NY; ^4^NYU Grossman School of Medicine, New York City, NY; ^5^Division of Biostatistics, NYU Grossman School of Medicine, New York City, NY; ^6^Division of Maternal‐Fetal Medicine, Department of Obstetrics and Gynecology, NYU Grossman School of Medicine, New York, NY; ^7^Department of Obstetrics and Gynecology, NYU Grossman School of Medicine, New York City, NY
9:45 AM	81	**Maternal and Fetal Cell Dynamics in the Placenta in Complicated and Healthy Pregnancy**
		**Rachelly Normand^1^**; Paola A. Lopez‐Zapana^2^; Daehee Han^3^; Courtney Ambrose^4^; Roya Best^5^; Zhaojing Liu^2^; Elizabeth Tuttle^5^; Christopher Stueber^5^; Olyvia J. Jasset^2^; Laura Ibanez‐Pintor^2^; Caroline Bald^2^; Lydia L. Shook^6^; Douglas A. Lauffenburger^7^; Alexandra‐Chloé Villani^8^; Andrea G. Edlow^9^ ^1^Massachusetts General Hospital, Massachusetts General Hospital, MA; ^2^Vincent Center for Reproductive Biology, Massachusetts General Hospital, Boston, MA; ^3^Vincent Center for Reproductive Biology, Massachusetts General Hospital; Department of Obstetrics and Gynecology, MassGeneral Brigham; Division of Maternal‐Fetal Medicine; Harvard Medical School, Boston, MA; ^4^Center for Immunology and Inflammatory Diseases, Department of Medicine and Krantz Family Center for Cancer Research, Massachusetts General Hospital, Boston, MA; ^5^Massachusetts General Hospital, Boston, MA; ^6^Department of Obstetrics and Gynecology, MassGeneral Brigham; Division of Maternal‐Fetal Medicine; Harvard Medical School, Massachusetts General Hospital/Boston, MA; ^7^Department of Biological Engineering, Massachusetts Institute of Technology, Cambridge, MA; ^8^Center for Immunology and Inflammatory Diseases, Krantz Family Center for Cancer Research, Massachusetts General Hospital, Boston, MA; ^9^Department of Obstetrics and Gynecology, MassGeneral Brigham; Division of Maternal‐Fetal Medicine; Harvard Medical School, Boston, MA

**Table:** 

Friday, February 13, 2026 8:00 AM – 10:00 AM		
Time	Abstract	Oral Concurrent Session 8 – Hypertension
8:00 AM	82	**Prospective Evaluation of Platelet Activity and Aspirin Responsiveness in Pregnancy**
		**Christina A. Penfield^1^**; Ariel Schapp^2^; Elliot Luttrell‐Williams^3^; Anais Hausvater^3^; Andre A. Robinson^1^; Yuhe Xia^4^; Matthew Muller^3^; Madison S. House^5^; Valeryia Avtushka^1^; Lauren Kennedy^3^; Rafid Quadir^3^; Justin S. Brandt^6^; Gwendolyn P. Quinn^7^; Ashley S. Roman^6^; Dana R. Gossett^7^; Jeffrey S. Berger^3^ ^1^Division of Maternal‐Fetal Medicine, Department of Obstetrics and Gynecology, NYU Grossman School of Medicine, New York City, NY; ^2^Division of Hematology Department of Medicine, NYU Grossman School of Medicine, New York City, NY; ^3^Leon H. Charney Division of Cardiology, Department of Medicine, NYU Grossman School of Medicine, New York City, NY; ^4^Division of Biostatistics, NYU Grossman School of Medicine, New York City, NY; ^5^NYU Grossman School of Medicine, New York City, NY; ^6^Division of Maternal‐Fetal Medicine, Department of Obstetrics and Gynecology, NYU Grossman School of Medicine, New York, NY; ^7^Department of Obstetrics and Gynecology, NYU Grossman School of Medicine, New York City, NY
8:15 AM	83	**Association of First Trimester Glycemia With Hypertensive Disorders of Pregnancy**
		**Lynn M. Yee^1^**; Nicola Lancki^2^; Patrick M. Catalano^3^; Francesca Facco^4^; Maisa Feghali^5^; William A. Grobman^6^; Erin S. LeBlanc^7^; William L. Lowe^8^; Audrey Merriam^9^; Mirella Mourad^10^; Caryn E.S. Oshiro^11^; Camille E. Powe^12^; Uma M. Reddy^13^; Jennifer Sherr^14^; Alexandra C. Spadola^15^; Kimberly K. Vesco^7^; Erika F. Werner^15^; Noelia Zork^13^; Denise Scholtens^1^; Dwight J. Rouse^16^ ^1^Northwestern University Feinberg School of Medicine, Chicago, IL; ^2^Northwestern University, Chicago, IL; ^3^MGH REU, Boston, MA; ^4^UPMC, UPMC, PA; ^5^Magee Women's Research Institute, University of Pittsburgh, Pittsburgh, PA; ^6^Warren Alpert Medical School of Brown University, Providence, RI; ^7^Kaiser Permanente Center for Health Research, Portland, OR; ^8^Northwestern University, Northwestern University, IL; ^9^Yale School of Medicine, Yale School of Medicine, CT; ^10^Division of Maternal‐Fetal Medicine, Department of Obstetrics and Gynecology, Columbia University Irving Medical Center, New York, NY; ^11^Kaiser Permanente Hawaii, Center for Integrated Health Care Research, Honolulu, HI; ^12^Massachusetts General Hospital, Boston, MA; ^13^Columbia University Irving Medical Center, New York, NY; ^14^Yale School of Medicine, New Haven, CT; ^15^Tufts University School of Medicine, Boston, MA; ^16^Women & Infants Hospital of Rhode Island/Warren Alpert Medical School of Brown University, Providence, RI
8:30 AM	84	**Multimodal Health System Intervention for Postpartum Hypertension: Influence on Readmission Rates**
		**Claire R. Conklin^1^**; Karen L. Li^2^; Jane W. Liang^3^; Monique Hedderson^4^; Susanna D. Mitro^5^; Mara B. Greenberg^6^ ^1^Kaiser Permenente, Kaiser/san Francisco, CA; ^2^Kaiser Permenente, Kaiser/northern california, CA; ^3^Kaiser Permenente, Kaiser/norther California, CA; ^4^Kaiser Permenente, Kasier/Northern California, CA; ^5^Kaiser Permenente, Kaiser/Norther California, CA; ^6^Kaiser Permenente, Kaiser/Oakland, CA
8:45 AM	85	**CATAlytic Antagonist of polyCOMB (CATACOMB) As a Master Regulator of the Placental Epigenome in Preeclampsia**
		**Sydney E. Dautel^1^**; Guomao Zhao^1^; Meera M. Thakkar^1^; Sunayana Srinivasan^1^; William E. Ackerman, IV^2^; Catalin S. Buhimschi^1^; Andrea Piunti^3^; Irina A. Buhimschi^2^ ^1^University of Illinois Chicago, University of Illinois Chicago, IL; ^2^University of Illinois Chicago, Chicago, IL; ^3^University of Chicago, University of Chicago, IL
9:00 AM	86	**Using Machine Learning to Predict Pre‐Eclampsia Before 20 Weeks Gestation**
		**Lauren M. Kucirka^1^**; Mohammad Kibria^2^; Leyu Dai^2^; Safoora Masoumi^2^; Mian Wei^3^; Metin Gurcan^4^; David Page^3^; Alison M. Stuebe^5^ ^1^UNC‐Chapel Hill, Chapel Hill, NC; ^2^University of North Carolina, Chapel Hill, NC; ^3^Duke University, Durham, NC; ^4^Wake Forest University, Winston‐Salem, NC; ^5^University of North Carolina at Chapel Hill School of Medicine, Chapel Hill, NC

**Table:** 

Friday, February 13, 2026 8:00 AM – 10:00 AM		
Time	Abstract	Oral Concurrent Session 8 – Hypertension, continued
9:15 AM	87	**A Randomized Controlled Trial for Postpartum Preeclampsia Antihypertensive Treatment (PPAT)**
		**Nadine Sunji^1^**; Amy Y. Pan^1^; Zaira Peterson^1^; Eleanor Saffian^1^; Alyssa M. Hernandez^1^; Anna Palatnik^2^ ^1^Medical College of Wisconsin, Medical College of Wisconsin, WI; ^2^Medical College of Wisconsin, MILWAUKEE, WI
9:30 AM	88	**Cost‐Effectiveness of Serial Laboratory Monitoring During Expectant Management of Early Preeclampsia With Severe Features**
		**Zakaria Doughan^1^**; Yossi Bart^2^; Joe Haydamous^3^; Wissam Akkary^2^; Ahmed Zaki Moustafa^4^; Baha M. Sibai^2^ ^1^Department of Obstetrics and Gynecology, McGovern Medical School at UT Health, Houston, Houston, TX; ^2^Division of Maternal‐Fetal Medicine, Department of Obstetrics, Gynecology and Reproductive Sciences, McGovern Medical School at UTHealth Houston, Houston, TX; ^3^Department of Obstetrics, Gynecology and Reproductive Sciences, McGovern Medical School at UTHealth Houston, Department of Obstetrics and Gynecology, McGovern Medical School at UT Health, Houston, TX; ^4^Division of Maternal‐Fetal Medicine, Department of Obstetrics, Gynecology and Reproductive Sciences, McGovern Medical School at UTHealth Houston, University of Texas ‐ Houston, TX
9:45 AM	89	**Adolescent Pregnancy and Increased Risk of Stroke: A Prospective Population‐Based UK Biobank Study**
		**Haemin Kim^1^**; Mi‐Young Oh^2^; Sang‑Hyuk Jung^3^; Young Mi Jung^4^; Manu Shivakumar^3^; Dokyoon Kim^3^; Ji Hoi Kim^5^; Jeesun Lee^6^; So Hee Kim^5^; Chan‐Wook Park^7^; Joong Shin Park^7^; Seung Mi Lee^8^ ^1^Kyungpook National University Chilgok Hospital, Daegu, Taegu‐Jikhalsi; ^2^Bucheon Sejong Hospital, Bucheon, Kyonggi‐do; ^3^University of Pennsylvania, Philadelphia, PA; ^4^Department of Obstetrics and Gynecology, Seoul National University Bundang Hospital, Seongnam‐si, Kyonggi‐do; ^5^Seoul National University Hospital, Seoul, Seoul‐t'ukpyolsi; ^6^Gyeongsang National University Hospital, Jinju‐si, Kyongsang‐namdo; ^7^Department of Obstetrics and Gynecology, Seoul National University Hospital, Seoul National University College of Medicine, Seoul, Republic of Korea, Seoul, Seoul‐t'ukpyolsi; ^8^Department of Obstetrics and Gynecology, Seoul National University College of Medicine, Seoul, Seoul‐t'ukpyolsi

**Table:** 

Friday, February 13, 2026 8:00 AM – 10:00 AM		
Time	Abstract	Oral Concurrent Session 9 – Quality
8:00 AM	90	**Reducing Obstetric Treatment Delays With Real‐Time Clinical Decision Support on Labor and Delivery**
		**Nandini Raghuraman^1^**; Megan L. Lawlor^2^; Lori M. Stevenson^3^; Steve Porter^4^; Susan Mann^5^; Amanda C. Zofkie^2^; Jeannie C. Kelly^1^; Antonina I. Frolova^2^; Roxane Rampersad^2^ ^1^Washington University School of Medicine, Washington University School of Medicine, MO; ^2^Washington University School of Medicine, St. Louis, MO; ^3^Barnes‐Jewish Hospital, St. Louis, MO; ^4^Case Western Reserve University, University Hospitals MacDonald Women's Hospital, Cleveland, OH; ^5^Beth Israel Deaconess Medical Center, Harvard Medical School, Boston, MA
8:15 AM	91	**Postpartum Hemorrhage Trends and Outcomes Before, During, and After a Statewide Obstetric Quality Improvement Effort**
		**Ellen Winter^1^**; Timothy Wen^2^; Mary E. D'Alton^3^; Dena Goffman^1^; Alexander M. Friedman^1^ ^1^Columbia University, New York, NY; ^2^UCSD, Irvine, CA; ^3^Division of Maternal‐Fetal Medicine, Department of Obstetrics and Gynecology, Columbia University Irving Medical Center, New York, NY
8:30 AM	92	**The Impact of an Inpatient MFM Telemedicine Program on Maternal Morbidity**
		**Adina R. Kern‐Goldberger^1^**; Cecilia Ganduglia Cazaban^2^; Chau Truong^2^; Sulki Park^2^; Sina Haeri^3^; Sindhu K. Srinivas^4^ ^1^Cleveland Clinic Lerner College of Medicine, Cleveland, OH; ^2^UTHealth Houston School of Public Health, Houston, TX; ^3^Ouma Health, Austin, TX; ^4^Department of Obstetrics and Gynecology, Perelman School of Medicine, Maternal and Child Health Research Center, Philadelphia, PA
8:45 AM	93	**Higher Hospital Volume Is Associated With Reduced Maternal Morbidity in Placenta Accreta Spectrum**
		**May Abiad^1^**; Scott A. Shainker^2^; Karin A. Fox^3^; Anna M. Modest^4^; Giulia Bonanni^5^; George R. Saade^6^; Hiba J. Mustafa^7^; Lamia K. Atasi^8^; Albaro Nieto‐Calvache^9^; Rachel K. Harrison^10^; J. M. Newton^11^; Andrea L. Greiner^12^; Sonya S. Abdel‐Razeq^13^; Daniela A. Carusi^14^; Alissa R. Carver^15^; Christina M. Duzyj^14^; Brett D. Einerson^16^; Clarel Antoine^17^; Julie Kang^18^; Deirdre J. Lyell^19^; Amir A. Shamshirsaz^20^; Alfred Abuhamad^6^; Baha M. Sibai^21^; Robert M. Silver^16^; Alireza A. Shamshirsaz^22^ On behalf of the PAN‐AMERICAN SOCIETY FOR THE PLACENTA ACCRETA SPECTRUM (PAS2) ^1^Baptist Memorial Medical Education, Memphis, TN; ^2^Department of Obstetrics and Gynecology, Beth Israel Deaconess Medical Center, Harvard Medical School, Boston, MA; ^3^Division of Maternal Fetal Medicine, The University of Texas Medical Branch Health, Galveston, TX; ^4^Beth Israel Deaconess Medical Center, Boston, MA; ^5^Boston Children's Hospital, Boston, MA; ^6^Eastern Virginia Medical School at Old Dominion University, Norfolk, VA; ^7^Division of Maternal‐Fetal Medicine, Indiana University School of Medicine, Indianapolis, IN; ^8^Division of Maternal‐Fetal Medicine, Department of Obstetrics and Gynecology, Mercy Hospital, St. Louis, MO; ^9^Departamento de Ginecología y Obstetricia, Unidad de Alta Complejidad Obstétrica, Fundación Clínica Valle del Lili, Cali, Valle del Cauca; ^10^Department of Maternal‐Fetal Medicine, Advocate Aurora Health, Chicago, IL; ^11^Division of Maternal‐Fetal Medicine, Department of Obstetrics and Gynecology, Vanderbilt University Medical Center,, Nashville, TN; ^12^Department of Obstetrics and Gynecology, University of Iowa Hospitals and Clinics, Iowa City, IA; ^13^Division of Maternal‐Fetal Medicine, Department of Obstetrics, Gynecology, and Reproductive Sciences, Yale University, New Haven, CT; ^14^Department of Obstetrics and Gynecology, MassGeneral Brigham; Division of Maternal‐Fetal Medicine; Harvard Medical School, Boston, MA; ^15^Department of Obstetrics and Gynecology, Wilmington Maternal‐Fetal Medicine, Wilmington, NC; ^16^Department of Obstetrics and Gynecology, University of Utah Health, Salt Lake City, UT; ^17^Department of Obstetrics and Gynecology, New York University Grossman School of Medicine, New York, NY; ^18^Department of Maternal‐Fetal Medicine, Memorial Healthcare System, Tampa, FL; ^19^Division of Maternal‐Fetal Medicine, Department of Obstetrics and Gynecology, Stanford School of Medicine, Stanford, CA; ^20^Division of Maternal‐Fetal Medicine, Department of Obstetrics and Gynecology, Baylor College of Medicine, Houston, TX; ^21^Division of Maternal‐Fetal Medicine, Department of Obstetrics, Gynecology and Reproductive Sciences, McGovern Medical School at UTHealth Houston, Houston, TX; ^22^Boston Children's Hospital, Brigham and Women's Hospital, Beth Israel Deaconess Medical Center, Harvard Medical School, Boston, MA

**Table:** 

Friday, February 13, 2026 8:00 AM – 10:00 AM		
Time	Abstract	Oral Concurrent Session 9 – Quality, continued
9:00 AM	94	**Maternal Vaccination Messaging: An Analysis of Patient‐Facing Web‐Based Informatics Using Advanced Data Scraping Technologies**
		**Marta Szymanska^1^**; Hanna R. Perone^2^; Kara Patek^3^; Bernard Gonik^4^ ^1^Corewell Health West, Holland, MI; ^2^Detroit Medical Center, Detroit, MI; ^3^Wayne State University Detroit Medical Center, Detroit, MI; ^4^Division of Maternal Fetal Medicine, Department of Obstetrics and Gynecology, Wayne State University, Detroit, MI
9:15 AM	95	**Virtual Maternity Program Engagement and Neonatal Outcomes: A Medicaid Dose‐Response Analysis**
		Lena Bertozzi^1^; Tanya Rudakevych^1^; Thomas Galeon^1^; Adam Kubsh^1^; Madeline Perry^2^; **Isabelle Von Kohorn^1^**; Ian J. Hooley^1^ ^1^Pomelo Care, Pomelo Care, NY; ^2^University of Pennsylvania, University of Pennsylvania, PA
9:30 AM	96	**Non‐Birthing Partner Outcomes in a Randomized Controlled Trial of the Baby2Home Digital Mental Health Intervention**
		**Emily S. Miller^1^**; Jacqueline Gollan^2^; Lutfiyya N. Muhammad^3^; Kathleen O'Sullivan^2^; Dinah Williams^4^; Malika D. Shah^3^; Lynn M. Yee^3^; Siyuan Dong^3^; Young S. Lee^3^; Craig F. Garfield^2^ ^1^Women & Infants Hospital of Rhode Island/Warren Alpert Medical School of Brown University, Providence, RI; ^2^Northwestern University Feinberg School of Medicine, Evanston, IL; ^3^Northwestern University Feinberg School of Medicine, Chicago, IL; ^4^Women & Infants Hospital of Rhode Island, Providence, RI
9:45 AM	97	**Maternal Outcomes After Implementation of a State Medicaid Agency and Hospital System Partnership Care Model**
		**Catherine Eppes^1^**; Suzanne Lundeen^2^; Ashley Sperber^2^; Sarah Detlefs^1^; Catherine Gannon^1^; Michelle Hansford^3^; Vaughan Gilmore^4^; Abigail Candelari^1^; Nidal Moukaddam^1^; Andres Ojeda^1^ ^1^Baylor College of Medicine, Houston, TX; ^2^Harris Health System, Houston, TX; ^3^Santa Maria Hostel, Housotn, TX; ^4^Santa Maria Hostel, Houston, TX

